# Poster Session II - A262 INTESTINAL EPITHELIAL CELL-SPECIFIC DELETION OF JAK2 DISRUPTS GUT HOMEOSTASIS AND INCREASES INTESTINAL PERMEABILITY

**DOI:** 10.1093/jcag/gwaf042.262

**Published:** 2026-02-13

**Authors:** B D’Mello, D Rivera, S Rehal, E Pollock-Tahiri, D Dodington, K Wagner, C Streutker, M Woo

**Affiliations:** Temerty, University of Toronto, Toronto, ON, Canada; University Health Network, Toronto, ON, Canada; University Health Network, Toronto, ON, Canada; University Health Network, Toronto, ON, Canada; University Health Network, Toronto, ON, Canada; Wayne State University, Detroit, MI; University of Toronto, Toronto, ON, Canada; University Health Network, Toronto, ON, Canada

## Abstract

**Background:**

The intestinal epithelium is essential for barrier integrity and mucosal immune regulation. While Janus kinase (JAK)–STAT signaling is implicated in inflammatory bowel disease (IBD) and JAK inhibitors are increasingly used, the endogenous role of JAK2 in intestinal epithelial cells (IECs) remains poorly defined.

**Aims:**

To define the in vivo role of epithelial JAK2 in barrier function, immune regulation, and colitis susceptibility.

**Methods:**

IEC-specific Jak2 knockout (vJak2 KO) mice were generated using the Villin-Cre/loxP system. Barrier function was assessed by FITC-dextran permeability assay. Tight junction, cytokine, and cell cycle gene expression was measured by RT-PCR. Histopathology and neutrophil infiltration were evaluated in H&E-stained sections. Colitis susceptibility was tested using dextran sodium sulfate (DSS). Western blotting for total and phosphorylated STAT was performed with small intestine (SI) lysates.

**Results:**

vJak2 KO mice were born at expected ratios and maintained normal body weight. When examined at 12 weeks of age, they had developed shortened colons and mild SI inflammation. vJak2 KO mice showed increased intestinal permeability, reduced tight junction gene expression, elevated *Il13* and *Il17*, and reduced *Il1b* and *Il6*. Histology revealed neutrophil infiltration, villous blunting, and higher pathology scores in the SI without overt colitis. In a model of DSS-induced colitis, vJak2 KO mice exhibited modest, and dose-dependent exacerbation of weight loss. Western blot analysis of SI lysates revealed largely preserved pSTAT1/tSTAT1, pSTAT3/tSTAT3, and pSTAT5/tSTAT5, with a selective increase in pSTAT6/tSTAT6 in vJak2 KO mice.

**Conclusions:**

Epithelial Jak2 supports barrier integrity and regulates expression of genes involved in mucosal immune state and integrity under steady-state conditions. These findings indicate that broad JAK inhibition may compromise epithelial function in IBD, highlighting the need to clarify the specific roles of JAKs and STATs in gut homeostasis and disease towards optimal therapeutic targeting.

**Funding Agencies:**

CIHR

